# A systematic survey of the measures used to identify postoperative changes in language function following epilepsy surgery

**DOI:** 10.1002/epi4.70164

**Published:** 2025-10-21

**Authors:** Isha Puntambekar, Matthias Koepp, Fenglai Xiao, Sallie Baxendale

**Affiliations:** ^1^ UCL Queen Square Institute of Neurology London UK; ^2^ University College Hospitals NHS Foundation Trust London UK

**Keywords:** confrontation naming, follow‐up interval, language outcomes, reliable change, temporal lobe epilepsy, verbal fluency

## Abstract

Epilepsy surgery, particularly dominant temporal lobe resection, poses a significant risk of post‐surgical language decline. There is considerable heterogeneity in the language assessment protocols employed across epilepsy surgery centers. This in turn is reflected in the observed variability in the incidence of language decline reported in the literature. We systematically surveyed cognitive outcome literature to critically appraise the nature and frequency of language assessment in order to establish the parameters of the existing evidence base. We found that confrontation naming was the most frequently used paradigm to assess language function (96%), followed by verbal fluency (47%) and language comprehension (13%). Over 75% of studies reported outcomes within the first two years of surgery, and close to 50% used reliable change index (RCI) to measure pre–post change in function, which is considered to be the gold standard. The evidence base is currently saturated with short‐term post‐surgical language outcomes assessed at single‐word level. Future studies employing comprehensive language assessment, longer follow‐up intervals, and designs comparing different metrics of pre–post change in function are needed.

**Plain Language Summary:**

- Patients undergoing surgery for temporal lobe epilepsy often experience a post‐surgical decline in language functions.
- The degree and frequency of language decline reported in research literature are influenced by the protocols used to assess language functions.
- Upon reviewing language outcome studies, we note a predominance of naming outcomes at short follow‐up intervals.
- Future studies incorporating comprehensive language testing at longer follow‐up intervals are needed to better counsel epilepsy patients of their lifelong risks of language decline, and allow them to make an informed decision.

**Key points**

- Up to 54% of patients undergoing a temporal lobe resection may experience a post‐surgical decline in language functions.
- The degree and frequency of post‐surgical language decline reported in research literature are influenced by center‐specific choice of tests, re‐test intervals, and approaches to measure change in performance.
- Upon systematically surveying the post‐surgical language outcome literature in temporal lobe epilepsy, we note that the evidence base is currently saturated with short‐term post‐surgical language outcomes assessed at the single‐word level.
- Future studies incorporating comprehensive language testing at longer follow‐up intervals are needed to better counsel epilepsy patients about their lifelong risks of language decline.

## 1 INTRODUCTION

Temporal lobe epilepsy (TLE) is associated with a variety of cognitive sequelae, including language deficits.^1^ While structural and functional abnormalities in cortical networks subserving language have been postulated to contribute to these impairments,^2^ pharmacological and non‐pharmacological interventions are also known to exacerbate them. Epilepsy surgery is associated with a further risk of post‐surgical language impairment, with reliable naming declines reported after 17%–54% of left‐ and less than 10% of right temporal resections.^3^, ^4^ These typically present as difficulties in word retrieval as well as general semantic abilities, and have a negative impact on patients' quality of life.^5^, ^6^

Risk factors for post‐surgical naming decline include later age of epilepsy onset, older age at surgery, resection of the language‐dominant hemisphere, and higher preoperative language functioning.^4^, ^7^, ^8^ However, reported post‐surgical language outcomes are also influenced by the choice of tests, re‐test intervals, and approaches to measure change in performance, all of which vary considerably across centers. These factors are reflected in the variations reported in post‐surgical language outcomes. For example, a 2018 study^9^ from the United States reported a 26% naming decline 6–12 months after a left or right temporal resection on the Boston naming test (BNT),^10^ while a 2020 study^11^ from the United Kingdom reported a 13.1% naming decline 12 months after surgery on the Graded naming test (GNT).^12^ Both these studies used Reliable Change Indices (RCI)^13^ at an 80% confidence interval to define meaningful postoperative change in English‐speaking samples, but on different naming tests. The range only widens with additional methodological and linguistic variability; up to 7% of a Finnish‐speaking sample exhibited naming declines one year after temporal resections, with decline defined as more than one standard deviation (SD) drop on a Finnish object naming test,^14^ while as high as 41% of an Italian‐speaking sample exhibited naming declines on an Italian adaptation of the BNT when decline was defined using RCI90.^15^ These examples illustrate the importance of a methodological context for estimates of post‐surgical language decline.

In this systematic survey of the research literature, we critically appraise the nature and frequency of language assessment reported in epilepsy surgery studies. We present a synthesis of findings with respect to:

- Measures of language assessment
- Follow‐up intervals
- Operational definitions of cognitive outcome/change

This study is not intended to audit the language assessment protocols in routine clinical practice across epilepsy surgery centers, but to survey the evidence base generated by these centers and summarize their research practices from a methodological perspective. Similarly, a review of language localization and lateralization practices in epilepsy surgery centers is beyond the scope of this study. We aim to establish the parameters of the current evidence base in order to clarify the scope of inferences drawn from it. Additionally, we wish to identify points of saturation and research gaps to inform future cognitive outcome studies.

## 2 METHODS

The protocol for this review was registered on Open Science Framework (https://doi.org/10.17605/OSF.IO/WK65Q).

We conducted a systematic literature search using PubMed, Embase, and APA PsychInfo to identify articles reporting factors predicting post‐surgical cognitive outcomes in adults with pharmacoresistant TLE. The search was limited to English‐language articles with full texts available. Articles involving pediatric and/or extra‐temporal epilepsy patient populations, as well as those reporting predictors of epileptogenic zone localization, post‐surgical seizure, or psychiatric outcomes, were excluded. Additional articles were either identified using the reference lists of eligible studies and relevant systematic and non‐systematic review articles, through snowballing, or suggested during external review. A comprehensive list of search terms, inclusion, and exclusion criteria can be found in the protocol linked above.

**FIGURE 1:**
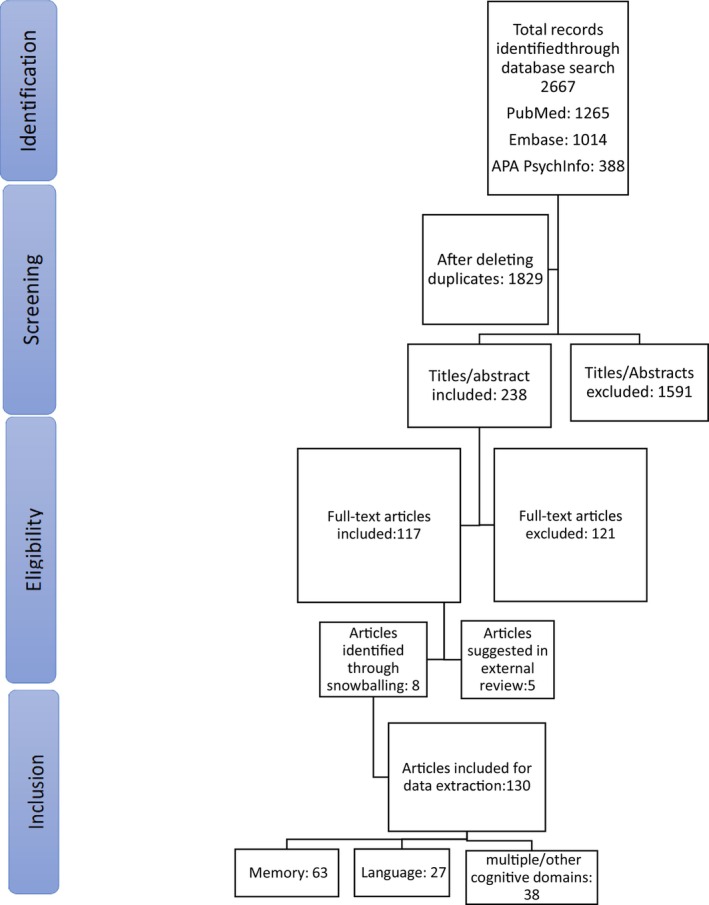
PRISMA flow diagram depicting study selection.

Data pertaining to the following variables of interest were extracted from the eligible studies: sample size and characteristics, neuropsychological domains assessed, neuropsychological tests used, test–retest interval, operational definitions of pre–post change in function. Additional information regarding the region of publication, the type of surgery, and the statistical approach used was also recorded (Figure 1).

## 3 RESULTS

This paper represents the first of a series appraising cognitive outcome literature from a methodological standpoint and covers language outcomes in detail. Other cognitive domains, including memory, will be covered in publications to follow.

Out of the 130 eligible studies, we identified 58 studies reporting postoperative language outcomes. Twenty‐seven studies investigated language as a primary outcome of interest, while the other 31 studies investigated language outcomes in combination with other cognitive domains. Sixty percent of the studies were in standard anterior temporal lobectomies with minor center‐specific variations, while the remainder included other surgery types such as lesionectomies, selective amygdalohippocampectomies, and more extensive temporal resections. The inconsistent level of detail across the included studies prevents us from presenting a formal classification.

Visual confrontation naming was the most frequently used paradigm to assess language function (55/58, 95%), followed by verbal fluency (26/58, 45%). Language comprehension tests were used less frequently (8/58, 14%). The BNT^10^ (35/55, 64%) was most commonly used to assess confrontation naming, while the Controlled Word Association test (COWAT)^16^ (9/26, 35%) and the FAS^17^ (4/26, 15%) test were commonly used to assess verbal fluency. The Token Test^18^ (7/8, 88%) was typically used to assess auditory language comprehension. Language measures in eight different languages across Europe and America were identified (Table 1).

**TABLE 1:** Language measures.

Test	Language	Frequency (%)
*Visual confrontation naming*		94.82
BNT	English^10^	47.27
	Italian^15^	5.45
	Spanish^19^	1.81
	Portuguese^20^	3.63
	German^21^, ^22^	5.45
GNT	English^12^	14.55
MAE‐VN+1	English^23^	7.27
AAT‐VN	German^24^	1.81
DO80	French^25^	5.45
Visual naming test	Dutch^26^	1.81
	English^27^	3.63
Object naming test	Finnish^28^	3.63
*Auditory confrontation naming*		3.44
Auditory naming test	English^27^	100
*Verbal fluency*		44.82
COWAT	English^16^	34.62
FAS	English^17^	15.39
Verbal fluency test (unspecified)	Italian^29^	11.54
	Portuguese^20^	3.84
	Czech^30^	3.84
	French^31^	11.53
	German^32^ Finnish^33^	15.38 3.84
*Auditory comprehension*		13.79
Token test	English^18^	25
	Italian^15^	12.5
	Finnish^34^	25
	German^24^	25
MAE‐aural comprehension	English^23^	12.5
*Aphasia screens*		3.44
Halstead Wepman aphasia screening exam	English^35^	100

Abbreviations: AAT, Aachener aphasia test; BNT, Boston naming test; COWAT, controlled word association test; DO80, test de dénomination orale d'images; FAS, Tombaugh FAS fluency; GNT, graded naming test; MAE, multilingual aphasia examination; VN, visual naming.

Forty‐nine studies reported cognitive outcomes at a single post‐surgical timepoint, with 41 reporting outcomes within the first two years of surgery. Only one study reported outcomes over 2 years post‐operatively. Nine studies reported outcomes at two or more surgical timepoints, and 4 of those 9 reported long‐term postoperative cognitive outcomes (Table 2).

**TABLE 2:** Follow‐up intervals.

Single time‐point (*n* = 49)										
≤6 m	≤12 m	≤24 m		>24 m		Unclear				
*n* = 14	*n* = 24	*n* = 3		*n* = 1		*n* = 7				
Multiple time‐points (*n* = 9)										
≤6 m	≤12 m	≤6 m	≤24 m	≤12 m	>24 m	≤6 m	≤12 m	≤24 m	>24 m	Unclear
*n* = 3		*n* = 1		*n* = 3		*n* = 1				*n* = 1

A variety of approaches were used to measure test–retest changes. Forty‐seven percent of the studies used RCI to define changes at the individual level in isolation (25/58, 43%) or in combination with other metrics such as SD (2/58, 7%). RCI90 was the most frequently used threshold. Thirty‐three percent of the studies used simple subtraction or absolute post‐operative scores, or conducted analyses at the group level only (Table 3).

**TABLE 3:** Approaches to measure language outcomes.

RCI/SRB	SD	Percentile/quartile	Combination	Absolute scores/group analyses	Unclear
43.10%	10.35%	5.17%	6.89%	32.76%	1.72%

Abbreviations: RCI, reliable change index; SD, standard deviation.

## 4 DISCUSSION

We systematically surveyed the methodological characteristics of the epilepsy surgery literature on language outcomes. Post‐surgical language outcomes were reported in close to 50% of studies that had looked at the impact of epilepsy surgery on cognitive function. A majority of these used visual confrontation naming tests in isolation to measure language outcomes within the first 2 years of surgery.

Our survey suggests that a preponderance of language outcome evidence in this context comes from short‐term post‐surgical naming outcomes. These outcomes are most commonly defined on the basis of RCI, and less frequently using SD, simple subtraction of absolute pre‐post scores, or change at group levels. Language domains such as spontaneous speech and verbal comprehension, as well as language outcomes beyond the first 2 years after temporal lobe resections, are rarely reported.

### 4.1 Language measures

#### 4.1.1 Confrontation naming

Word‐finding difficulties are a chief cognitive complaint among epilepsy patients with a seizure focus in the language‐dominant hemisphere. Moreover, they often reflect impairment on objective tests of naming.^36^ Visual confrontation naming tasks require participants to name pictures of objects on demand and are known to be sensitive to language‐dominant temporal dysfunction.^37^ BNT is the most widely used test of visual confrontation naming with established sensitivity and predictive value in the context of epilepsy surgery. Our findings reflect this; BNT was identified as the most common test of confrontation naming, with at least 4 other language adaptations. Other similar tasks of visual confrontation naming employed were the GNT,^12^ the DO80 and the Object naming test.^28^

It has been argued that auditory naming measures are more sensitive to word‐finding difficulties in TLE as they tap into retrieval mechanisms that are more ecologically relevant.^38^, ^39^ Moreover, temporal resections have distinct effects on auditory and visual naming functions,^40^, ^41^ with a qualitative report suggesting that the resection of extraoperatively mapped auditory naming sites leads to both visual and auditory naming declines despite the sparing of visual naming sites.^42^ However, only two studies under review reported auditory naming outcomes.^40^, ^42^

#### 4.1.2 Verbal fluency

Verbal fluency tests require the examinee to spontaneously produce words that meet certain task constraints (e.g., beginning with the given letter, belonging to a particular category), while suppressing irrelevant responses and avoiding repetition by maintaining the earlier responses in working memory. Thus, in addition to the obvious linguistic component, verbal fluency tasks also tap into the domains of cognitive flexibility, executive control, and working memory.

Depending on the task constraints, verbal fluency tests consist of two distinct subtypes: phonemic fluency tests require participants to generate words starting with a specified letter, excluding proper nouns (e.g., S). Semantic fluency tests require participants to generate words belonging to a specified semantic category (e.g., Animals). Production of multiple words in succession has been hypothesized to rely on the ability to produce clusters of phonemically related (e.g., words beginning with ‘st’ for the letter ‘S’) or semantically related words (e.g., domestic animals for the category ‘Animals’), and ‘switching’ from one cluster to the next once it is exhausted.^43^, ^44^, ^45^

COWAT^16^ and FAS^17^ emerged as the most commonly used fluency tests in English. We identified six more language‐specific fluency tests with little to no information about the phonemic cues or categories used. While a majority of these studies used a combination of categorical and phonemic fluency, six used one or the other.

Semantic fluency tasks are understood to rely more on semantic retrieval networks mediated by the temporal lobe, while phonemic fluency tasks are thought to be more dependent on frontally mediated strategic searches.^46^ However, both phonemic and semantic fluency impairments are reported in TLE patients. There is no evidence to indicate that one or the other fluency subtests is more sensitive to language or executive functioning impairments in TLE, which raises the possibility that the decisions to use semantic or categorical fluency tests in isolation may have been driven by data availability.

#### 4.1.3 Language comprehension

Visual and auditory language comprehension above the single‐word level involves both left and right temporal lobes and requires understanding of vocabulary and syntax, attention, and working memory abilities. The Token test^18^ is a test of auditory comprehension developed to diagnose receptive disturbances in patients with aphasia. The test requires the examinee to follow a series of commands pertaining to a number of tokens with different shapes and colors. We note 7 instances of the Token test being utilized in at least 4 linguistically distinct TLE surgical populations.

While the frequency profile for visual confrontation naming and verbal fluency is largely in agreement with the European survey of neuropsychology practices in epilepsy surgery centers,^47^ only 20% of the European studies identified in our survey reported outcomes in this domain, compared to 35% of European surgical centers reportedly using language comprehension tests such as the Token test in clinical practice. This difference suggests a potential discrepancy between clinical and research practices, whereby only a partial clinical battery seems to make its way into the centers' research output. One might speculate that this is a deliberate omission due to the evident ceiling effects in tests like the Token test or the low incidence of significant language comprehension deficits in the TLE population. Even if comprehension deficits are not apparent at the group level, individual patients with impaired language comprehension may warrant a closer look to rule out atypical language impairments. Furthermore, auditory comprehension was predominantly assessed using the Token test in the studies reviewed, with a single instance of the aural comprehension subtest of the MAE. While it is useful to screen gross comprehension deficits, a more nuanced assessment of language comprehension is warranted to reliably rule out deficits in this clinical population.

It is important to note here that the studies under review span epilepsy centres across the globe, but equivalent clinical practice surveys for non‐European countries are not currently available. Therefore, the inferences drawn here cannot be extended to clinical practices beyond Europe.

#### 4.1.4 Complete batteries and screens

We identified two instances of an aphasia screen being used; the Halstead–Wepman Aphasia screening test consists of a series of stimuli, the response to which is used to rapidly screen for language deficits, including agnosias, apraxias, anomias, dysarthrias, and paraphasias.^35^

Consistent with previous reports,^48^, ^49^ a majority of language outcome studies used visual confrontation naming or verbal fluency tests in isolation to infer general language functioning.^49^ Considering that naming deficits are the most salient language deficit in this population, it is not surprising that 62% of the included studies used a visual confrontation naming test as a sole language measure. However, multiple reports caution against the portrayal of ‘naming’ as a unitary entity, given the nuances associated with the modality of the naming paradigm in use.^38^, ^39^, ^40^ Furthermore, 10% of the included studies used verbal fluency tests in isolation. Since verbal fluency is far from a ‘pure’ test of language functioning, it is even less appropriate as a proxy measure for overall language functioning in TLE.

As TLE patients rarely present with aphasia, their general language comprehension abilities have not been of research interest. There is modest evidence to indicate language deficits in TLE patients extend beyond dysnomia and include subdomains of receptive language and spontaneous speech production.^48^, ^50^, ^51^, ^52^ The effect of surgery on these abilities is not clear. Moreover, the integrity of basic language functions also contributes to the adequacy of verbal memory,^52^, ^53^, ^54^, ^55^ which is of considerable clinical and research interest. While a majority of research investigating this contribution has focused on visual naming,^52^, ^54^, ^55^ a 1988 study by Hermann and colleagues highlights the contribution of auditory and reading comprehension as well as word association impairments to deficits on various California Verbal Learning test subtests. It is not a stretch to imagine that these deficits, while less salient than dysnomia, might also make themselves known in patients' day‐to‐day interactions, and therefore affect their quality of life. A data mining study unveiled concerns related to reading, writing, and spelling in addition to word finding and verbal memory among adult epilepsy patients and caregiver populations.^56^ The authors further noted a mismatch between the extant research literature focusing on single‐word productions and the rare assessment of higher‐level language and discourse abilities and subjective patient concerns retrieved from social media.

A good example of a higher‐level language deficit in TLE is a characteristic, verbose, and pedantic speaking style termed ‘circumstantiality of speech’. Frequently reported by TLE patients and clinicians alike, circumstantiality of speech has been hypothesized to compensate for reduced memory capacity as opposed to deficient lexical retrieval.^50^, ^51^ Recent work analyzing unstructured discourse production in TLE patients revealed a pattern wherein patients produced longer, repetitive, less concise, less fluent, and less informative output, generally ‘saying more about less’,^57^, ^58^ a feature that did not improve over repetitions.^58^ Neurolinguistic deficits such as circumstantiality are unlikely to come to light in standardized neuropsychological assessment but warrant further research into their neuropsychological correlates and possible implications on social and occupational functioning. One promising avenue in this regard is spontaneous speech analysis using automated feature extraction and machine learning‐ or deep learning‐based classification models, which has garnered some support in Alzheimer's disease literature as a reliable digital biomarker.^59^, ^60^, ^61^ A similar technique may be feasible for routine use in the epilepsy clinic, whereby spontaneous speech samples recorded as a part of the clinical interview may be used to extract nuanced linguistic features that may be missed by the prevalent assessment protocols.

### 4.2 Different languages

The studies reviewed here employed language measures spanning eight languages across Europe and America. These included both adaptations of measures originally normed in English as well as original tests developed and normed locally. Language outcome studies from Asia and other parts of the world were notably absent from the review, which has important implications for the generalizability of any inferences drawn from this literature. Cognitive consequences of epilepsy surgery may be more favorably biased due to the overrepresentation of European and American patients with access to the best care.^62^

Neuropsychology is an emerging discipline in various stages of development across low‐ and middle‐income countries, which no doubt explains the paucity of research literature from these regions. For example, a recent survey from India highlighted that the limited availability of language‐specific standardized tests with current norms was an important barrier to neuropsychological input in surgical management.^63^

The implications of a linguistically unrepresentative evidence base extend also to the linguistic minorities in Western countries. Language discordance is widely acknowledged as a barrier to healthcare access, quality, and research participation.^64^, ^65^ A retrospective cohort study from the University of California found that limited English proficiency was associated with significantly lower odds of undergoing an anterior temporal resection for unilateral mesial temporal sclerosis.^65^ In the absence of representative cognitive outcome data, these patients cannot be satisfactorily counseled about the cognitive risks of epilepsy surgery.

### 4.3 Follow‐up interval

Epilepsy surgery confers cognitive risks over and above the epilepsy itself. Surgery in general, as well as distinct aspects of the procedure, including anesthesia and hospitalization, have been viewed to be akin to a neurophysiological “stress test” for the brain, often associated with perioperative cognitive sequela.^66^ In the context of epilepsy, the surgical resection may be conceptualized as a controlled traumatic brain injury and impacts cognitive functions in a similar manner. Organic brain recovery following a resective surgery must therefore be factored into the follow‐up interval.

The first year after surgery has been the focus of cognitive outcome literature more generally. This is reflected in our results: 78% of the studies only report outcomes within the first two years of surgery. However, the need for long‐term cognitive outcomes is being increasingly recognized. We identified five studies reporting outcomes at 3,^14^ 4,^67^ 5 years,^15^, ^30^ and 9 years^68^ after surgery. Studies that have employed longitudinal designs with outcomes measured at multiple post‐operative time points report dynamic changes in function that cannot be captured at a single short‐term post‐surgical time point when cognitive functions are yet to stabilize.^69^ While language functions are known to be robust to age effects in the healthy population,^70^, ^71^ the available evidence is not sufficient to say whether this holds true in patients with TLE; it is not clear how surgical lesions interact with longitudinal age‐ and disease‐related changes in language functioning in these patients.

Within the existing long‐term studies, language outcomes were assessed using verbal fluency test (4/5), visual naming (2/5), language comprehension (1/5), and WAIS language subtests in 5 different languages, namely English, Italian, German, Finnish, and Turkish. Four out of five studies reported individual‐level change using RCI or SD, while one reported group‐level analyses only. Age at long‐term follow‐up was less than 60 in all studies. Therefore, the existing evidence does not cover the entire breadth of language functions nor the lifespan. While these studies reported stable or improved language function for a majority of the patients,^14^, ^15^, ^72^ it is not clear if these gains are maintained throughout the lifespan, in the face of aging, fluctuations in seizure control, and antiseizure medication use. This information is crucial for counseling patients about their lifelong cognitive risks of epilepsy surgery and allowing them to make informed decisions. Further longitudinal cognitive outcome research with longer follow‐up intervals is necessary, also to better characterize the risk factors for the subgroup of patients with persistent language deficits or delayed declines.

### 4.4 Measurement of change

In addition to aiding in the localization of the seizure focus, pre‐surgical neuropsychological assessment of language helps establish a presurgical baseline to measure future change against, and estimate the risk of postsurgical language decline by predictive modeling. The clinical utility of predictive models to estimate this risk depends on the definition of change used.

The present review indicates a move towards individual level analyses with predefined cut‐offs based on RCI, SD, or percentiles to measure clinically significant change. RCI was by far the method of choice reported in 43% of the studies, with variable stringencies ranging from 80% to 95% confidence interval. Three studies reported the use of Standardized Regression‐based norms (SRB). All of these were published within the last 25 years, the oldest one dating back to 1996. Ten percent used SDs to define change and predominantly used 1SD as the stringency, while only one study used 1.5SD. Thirty‐three percent of the studies defined post‐surgical language outcomes using absolute scores or simple subtraction. A variety of factors, such as lack of published RCI cut‐offs for the test in use or simply ease of measurement, may explain this.

Selecting an appropriate definition of change involves multiple considerations. Approaches such as simple subtraction of raw scores fail to take into account the clinical significance of the observed changes. Raw scores are highly susceptible to practice effects, which may artificially inflate test scores, leading to them being clinically misinterpreted as stable or improved from baseline. SD and percentile changes offer a modest advantage over these methods in that they make use of standardized scores as opposed to raw scores, but their chosen thresholds of stringency are highly heterogeneous and often arbitrary. These approaches are additionally confounded by issues of test–retest reliability and regression to the mean.^13^, ^73^ RCI and SRB are robust against practice effects, regression toward the mean, and take into account the test–retest reliability to produce thresholds for clinically meaningful change. However, the heterogeneity in stringency of cut‐offs persists, albeit less arbitrary than that observed with SD and percentiles.

The generalizability as well as clinical utility of the aforementioned outcome prediction models is contingent upon the harmonization of the operational definition of change. Studies comparing two or more approaches to define change, as well as different stringencies within the same approach to assess their relative performance, would be a necessary first step in that direction.

## 5 LIMITATIONS

We systematically reviewed postoperative cognitive outcome literature in TLE and present a survey of language assessment protocols reported within this literature. As our search terms were designed to pick up pre‐post studies predicting postoperative cognitive outcomes, they may have failed to flag language‐specific studies evaluating different language assessments or cortical language mapping protocols. While these studies fall within the scope of the survey, the slight discrepancy in their primary aims and that of the review may have led to their omission. We are grateful to the reviewers for their assistance in identifying some of the studies missed in the original search.

## 6 CONCLUSIONS

We have presented the findings of a systematic survey of the methods used to assess language outcome following epilepsy surgery. We cataloged language batteries in 8 different languages across Europe and America that largely coincide with clinical practice. Most of the reviewed studies reported individual‐level analysis, and about half adhered to gold‐standard approaches such as RCI to measure pre–post change in function. However, the evidence base is currently saturated with short‐term post‐surgical language outcomes assessed at the single‐word level of function.

Future research should focus on (1) a more comprehensive assessment of language functions, (2) follow‐up intervals beyond the first two years after surgery, and (3) individual‐level designs comparing different approaches to define pre–post change in function.

## AUTHOR CONTRIBUTIONS

**Isha Puntambekar:** Data acquisition, analysis, manuscript writing, and editing. **Sallie Baxendale:** Conceptualization, study design, supervision, manuscript review, and feedback. **Matthias Koepp:** supervision, manuscript review, and feedback. **Fenglai Xiao:** manuscript review and feedback.

## FUNDING INFORMATION

This work is supported by the Wellcome Trust. Grant number: 221934/Z/20/Z. Grant name: Joint Investigator Award in Science, ‘Epilepsy and neurodegeneration: disease mechanisms and early detection’.

## CONFLICT OF INTEREST STATEMENT

The authors declare no conflict of interest. We confirm that we have read the Journal's position on issues involved in ethical publication and affirm that this report is consistent with those guidelines.

## Supporting information


**Table S1.** List of studies under review.

## Data Availability

Not applicable. Review of published literature.
