# Supplementary material for: A systematic survey of the measures used to identify postoperative changes in language function following epilepsy surgery
Source: Epilepsia Open. 2025 Oct 21;10(6):1751–61. doi: 10.1002/epi4.70164 (PMC12716311; doi:10.1002/epi4.70164)
Supplement: Supplementary file 1 — Table S1. List of studies under review. [file EPI4-10-1751-s001.docx]

A systematic survey of the measures used to identify postoperative changes in language function following epilepsy surgery.

Supplementary table 1. List of studies under review

| Studies | Language tests used |
| --- | --- |
| Davies, Risse & Gates (2005) | BNT |
| Giovagnoli et al. (2016) | BNT, verbal fluency, token test |
| Gross et al. (2022) | BNT |
| You et al. (2019) | BNT |
| Trimmel et al. (2019) | GNT |
| Busch et al. (2018) | BNT |
| Reindl et al. (2022) | BNT |
| Osipowicz et al. (2016) | COWAT semantic fluency |
| Schwarz, Pauli & Stefan (2005) | BNT |
| Rosazza et al. (2013) | BNT, Verbal fluency |
| Langfitt & Rausch (1996) | BNT |
| Davies et al. (1998) | BNT, MAE- VN, Token test, COWAT |
| Abdallah et al. (2021) | DO80, Verbal fluency |
| Escorsi-Rosset et al. (2011) | BNT |
| Schwartz et al. (1998) | BNT |
| Stafiniak et al. (1990) | BNT |
| Kovac et al. (2010) | BNT |
| Ruff et al. (2007) | BNT |
| Yogarajah et al. (2010) | GNT, Tombaugh verbal fluency |
| Powell et al. (2008) | GNT |
| Foesleitner et al. (2021) | BNT, Regensberg verbal fluency test |
| Bonelli et al. (2021) | GNT, Verbal fluency test |
| Janecek et al. (2013) | BNT |
| Sabsevitz et al. (2003) | BNT |
| Binding et al. (2023) | GNT |
| Prada Jardim et al. (2017) | GNT |
| Kamm et al. (2017) | BNT, COWAT |
| Cano-Lopez et al. (2017) | BNT, Verbal fluency |
| Rausch et al. (2003) | WAIS Vocabulary and Similarities |
| Vogt et al. (2018) | BNT, Token test, Semantic fluency |
| Doucet et al. (2015) | BNT |
| Baxendale & Thompson (2020) | GNT, Verbal fluency |
| Pauli et al. (2017) | BNT, Verbal fluency |
| Liu et al. (2017) | BNT |
| Mathon et al. (2017) | DO80, Verbal fluency |
| Jutila et al. (2014) | Object naming test, Verbal Fluency Test, Token test |
| Clearly et al. (2013) | GNT, Category fluency |
| Clusmann et al. (2002) | Aachen Aphasie test- verbal fluency and naming subtests, Token test |
| Potter et al. (2009) | BNT, MAE- VN and COWAT subtests |
| Paff et al. (2021) | BNT |
| Hermann et al. (1991) | MAE |
| Cheluene et al. (1990) | BNT, COWAT, Halstead Wepman Aphasia Screening exam, Speech sounds perception test |
| Tuunainern et al. (1995) | Object naming test, Token test |
| Morita-Sherman et al. (2020) | BNT |
| Ojemann et al. (1985) | Halstead Wepman Aphasia Screening exam |
| Bell et al. (2000) | BNT, MAE-VN subtest |
| Sever et al. (2018) | BNT, COWAT |
| Uijil et al. (2009) | Visual naming task |
| Roger et al. (2021) | DO80, Verbal fluency |
| Hinds et al. (2023) | BNT, COWAT Semantic fluency |
| Giovagnoli et al. (2016) | BNT |
| Helmstaedter et al. (2003) | Phonemic fluency |
| Kramska et al. (2017) | Verbal fluency |
| Hamberger et al., (2005) | BNT, VNT, ANT |
| Hamberger et al., (2010) | BNT, VNT, ANT |
| Drane et al., (2015) | BNT |
| Martin et al., (2000) | COWAT phonemic fluency test |
| Seidenberg et al., (2010) | MAE-VN & Aural comprehension |
